# Intra-articular Entrapment of Medial Epicondyle Fracture Fragment in Elbow Joint Dislocation Causing Ulnar Neuropraxia: A Case Report

**DOI:** 10.5704/MOJ.1703.016

**Published:** 2017-03

**Authors:** J Syed, AR Zamri, S Jamaluddin, JK Ruben, M Gopindran

**Affiliations:** Department of Orthopaedics, Kuala Lipis Hospital, Kuala Lipis, Malaysia

**Keywords:** paediatric elbow dislocation, incarcerated medial epicondyle, paediatric ulnar neuropraxia

## Abstract

Traumatic elbow dislocations in children are rare but most of them are complex dislocations, and in such dislocations, medial humerus epicondyle fractureis the most common associated injury. Fracture incarceration in the elbow joint occurs in 5-18% of medial humerus epicondyle fractures but ulnar neuropraxia is very rare. Open reduction internal fixation is indicated in medial humerus epicondyle fracture with fracture incarceration, ulnar neuropraxia, marked instability or open fracture. Operative treatment options include fragment excision and sutures, closed or open reduction and Kirschner wire fixation, open reduction and suture fixation, open reduction and smooth pin fixation, and open reduction and screw fixation. However, ulnar nerve transposition is debatable as good outcome had been reported with and without nerve transposition. We report a case of a 13-year old boy, who presented with right elbow dislocation and intra-articular entrapment of medial humerus epicondyle fracture fragment, complicated with sensory ulnar neuropraxia, following a fall onto his right outstretched hand in a motor vehicle accident. The elbow joint was reduced using close manipulative reduction but the fracture fragment remained entrapped post-reduction. The patient then underwent open reduction and screw fixation of the medial humerus epicondyle fracture without ulnar nerve transposition. He had good functional outcome six weeks after surgical intervention, with complete recovery of ulnar neuropraxia six months later. Currently, he is doing well at school and is active with his sporting activity.

## Introduction

Traumatic elbow dislocation in children is rare with an incidence of 3-6% of all elbow injuries ^1^. Most paediatric elbow dislocations are complex dislocations and medial humerus epicondyle avulsion fracture is the most common associated injury^1^. In medial humerus epicondyle fracture, intra-articular entrapment of fracture fragment in the elbow joint occurs in 5-18% of cases^2^ but ulnar neuropraxia is very rare^1,3,4^. Treatment of such injury involves closed manipulative reduction of the elbow dislocation and open reduction and internal fixation of the fracture using various techniques which include screw fixation, Kirschner wiring, sutures or excision of the fracture fragment^2^. However, ulnar nerve transposition is still debatable^3-5^.

## Case Report

A 13-year old boy presented to our hospital with a history of fall onto his right outstretched dominant hand after being involved in a motor vehicle accident. He complained of pain, immediate swelling, and restricted range of motion of the right elbow with loss of sensation over the right ring and little fingers following trauma. Physical examination revealed deformity and generalized swelling of the elbow joint with tenderness at the medial joint line on palpation. Both active and passive elbow joint range of motion were restricted.

**Fig. 1:**
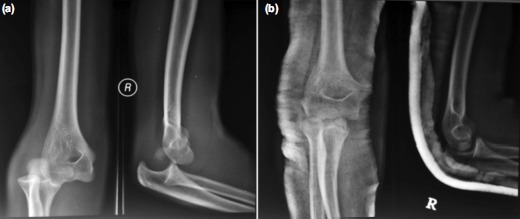
(a) Right elbow radiograph showing right posterolateral elbow dislocation with intraarticular entrapment of medial humerus epicondyle avulsion fracture fragment in the elbow joint. (b) Medial humerus epicondyle fracture fragment remained entrapped in the elbow joint after closed manipulative reduction.

Radiograph of the right elbow showed posterolateral elbow dislocation with intra-articular entrapment of the medial humerus epicondyle avulsion fracture fragment (Fig. 1a). Closed manipulative reduction under sedation was attempted and the elbow joint was successfully reduced. However, the medial humerus epicondyle fracture fragment remained entrapped in the elbow joint (Fig. 1b) and sensory ulnar neuropraxia persisted. Four days after the injury, the patient underwent open reduction and internal fixation of the medial humerus epicondyle avulsion fracture with ulnar nerve decompression through medial elbow approach.

**Fig. 2:**
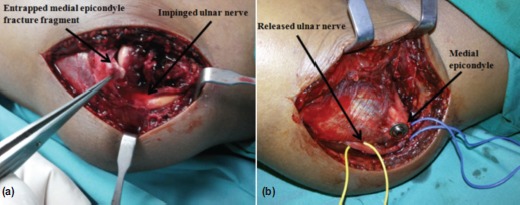
(a) Entrapped medial humerus epicondyle fracture fragment in the elbow joint impinging on the ulnar nerve. (b) Ulnar nerve explored and released but not transposed and screw fixation of the medial humerus epicondyle fracture fragment.

Intraoperatively, it was noted that the medial humerus epicondyle fracture fragment was entrapped in the elbow joint and impinged on the ulnar nerve distal to the Struther’s ligament (Fig. 2a). The fracture fragment was retrieved from the joint and fixed using two 4.0 mm cannulated cancellous screws directed perpendicular to the fracture line and anteriorly to avoid from entering the olecranon fossa. The ulnar nerve was decompressed but not transposed. It was covered with surrounding soft tissue to avoid irritation from the hardware (Fig. 2b).

**Fig. 3:**
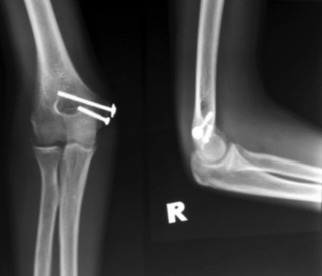
Radiographs showing united medial humerus epicondyle avulsion fracture.

Post-operatively the elbow was supported in a splint in 90 degree flexion. The wound healed well, and range of motion exercise was started two weeks after surgery. At six weeks follow up, fracture union was noted on radiography (Fig. 3) and the patient achieved full active elbow range of motion. Complete recovery of the ulnar sensory neuropraxia occurred six months after surgery.

## Discussion

Traumatic elbow dislocation in children is rare with an incidence of 3-6% of all elbow injuries^1^. Most of them are complex dislocations and posterolateral in direction ^1^. Associated injuries in complex paediatric elbow dislocation includes fractures of the medial humerus epicondyle, coronoid process, radial head, trochlea or lateral humerus epicondyle or disruption of the proximal radio-ulnar joint^1^. Medial humerus epicondyle fracture is the most common associated injury in complex paediatric elbow dislocation^1^. Intra-articular entrapment of the fracture fragment in the elbow joint occurs in 5-18% of cases ^2^ but ulnar neuropraxia is rare^1,3,4^. In children, the medial collateral ligament is intact during elbow dislocation contributing to medial humerus epicondyle fracture^2^. The ulnar nerve could be injured due to either direct compression from the fracture fragment as evident in our case or during elbow joint manipulation^4^.

Incarcerated epicondyle fracture fragment is difficult to visualise on radiograph as it can overlap with the distal humerus metaphysis or be confused with the ossification centres, thus further imaging is required ^4^. Failure to recognize this can result in restricted elbow mobility and increased ulnar nerve injury ^4^. Both conservative and operative treatment had been described for management of medial humerus epicondyle fracture ^2^. Currently, the absolute indications for open reduction and internal fixation include epicondyle fracture fragment incarceration in the elbow joint, ulnar neuropraxia, marked instability, and open.

The goal of operative treatment is to achieve stable fixation, thus allowing early elbow mobilization and preventing elbow deformity or stiffness ^2^. Operative treatment options include fragment excision and sutures, closed or open reduction and Kirschner wire fixation, open reduction and suture fixation, open reduction and smooth pin fixation, and open reduction and screw fixation^2^. Operative treatment with suture fixation is unstable thus immobilization with a splint is required post-operatively ^2^. Kirschner wire fixation provides greater stability over sutures but also requires postoperative immobilization ^2^. Furthermore, Kirschner wires tend to impinge onto the skin thus inhibiting early range of motion and causing inadequate compression which can lead to fracture nonunion^2^.

Open reduction and screw fixation provide stable fixation, 100% fracture union and excellent functional outcomes ^2^. However, screw prominence can cause irritation and interference with bone growth, thus implant removal is required at a later stage^2^. Fowles *et al* recommended anterior ulnar nerve transposition in patients with signs of ulnar nerve compression ^3^. However, a study by Chen *et al* showed that ulnar nerve transposition could increase the risk of ulnar neuritis^5^. In this case, open reduction and internal fixation of the medial humerus epicondyle fracture was performed as the fracture fragment was entrapped in the elbow joint causing sensory ulnar neuropraxia. Screw fixation was the method of choice as it could provide adequate compression of the fracture fragment thus allowing fracture union and early joint mobilization.

Ulnar nerve transposition was not performed in this case. In our opinion, the ulnar neuropraxia could resolve with reduction and fixation of the fracture as direct compression of the nerve by the fracture fragment was the cause of the neuropraxia. Furthermore, ulnar nerve transposition requires a longer incision, increases the risk of neuritis due to implant irritation and could cause nerve injury during implant removal. The functional outcome was good in this case. The child regained full active elbow range of motion six weeks post-operatively and complete recovery of ulnar neuropraxia six months later. Currently, he is doing well at school and is active with his sporting activity. We plan to remove the implant in one year’s time.
